# Revealing the static and dynamic nanomechanical properties of diatom frustules—Nature's glass lace

**DOI:** 10.1038/s41598-023-31487-x

**Published:** 2023-04-04

**Authors:** Julijana Cvjetinovic, Sergey Yu. Luchkin, Eugene S. Statnik, Nickolai A. Davidovich, Pavel A. Somov, Alexey I. Salimon, Alexander M. Korsunsky, Dmitry A. Gorin

**Affiliations:** 1 Center for Photonic Science and Engineering, Skolkovo Institute of Science and Technology, Bolshoy Boulevard 30, Bld. 1, 121205 Moscow, Russia; 2 Center for Energy Science and Technology, Skolkovo Institute of Science and Technology, Bolshoy Boulevard 30, Bld. 1, 121205 Moscow, Russia; 3 T.I. Vyazemsky Karadag Scientific Station, Natural Reserve of the Russian Academy of Sciences, Kurortnoe, 98188 Feodosiya, Russia; 4 College of New Materials and Nanotechnologies, National University of Science and Technology MISiS, 4, Leninskiy Prospekt, 119049 Moscow, Russia; 5 Department of Engineering Science, University of Oxford, Oxford, OX1 3PJ UK

**Keywords:** Biophysics, Biotechnology, Materials science

## Abstract

Diatoms are single cell microalgae enclosed in silica exoskeletons (frustules) that provide inspiration for advanced hybrid nanostructure designs mimicking multi-scale porosity to achieve outstanding mechanical and optical properties. Interrogating the structure and properties of diatoms down to nanometer scale leads to breakthrough advances reported here in the nanomechanical characterization of *Coscinodiscus oculus-iridis* diatom pure silica frustules, as well as of air-dried and wet cells with organic content. Static and dynamic mode Atomic Force Microscopy (AFM) and in-SEM nanoindentation revealed the peculiarities of diatom response with separate contributions from material nanoscale behavior and membrane deformation of the entire valve. Significant differences in the nanomechanical properties of the different frustule layers were observed. Furthermore, the deformation response depends strongly on silica hydration and on the support from the internal organic content. The cyclic loading revealed that the average compliance of the silica frustule is 0.019 m/N and increases with increasing number of cycles. The structure–mechanical properties relationship has a direct impact on the vibrational properties of the frustule as a complex micrometer-sized mechanical system. Lessons from Nature’s nanostructuring of diatoms open up pathways to new generations of nano- and microdevices for electronic, electromechanical, photonic, liquid, energy storage, and other applications.

## Introduction

Well-ordered nanostructured natural objects with advanced properties provide an unending source of inspiration for the development of new materials and devices in a widest range of applications. A rich gallery of such objects are the skeletons of diatom algae—Nature’s glass (silica) lace. The neat slender structures are remindful of woven lace that combines elegance with strength and durability, and provoke the effort to explore the organization and properties of the objects created and refined by nature over millions of years of evolution.

**Figure 1:**
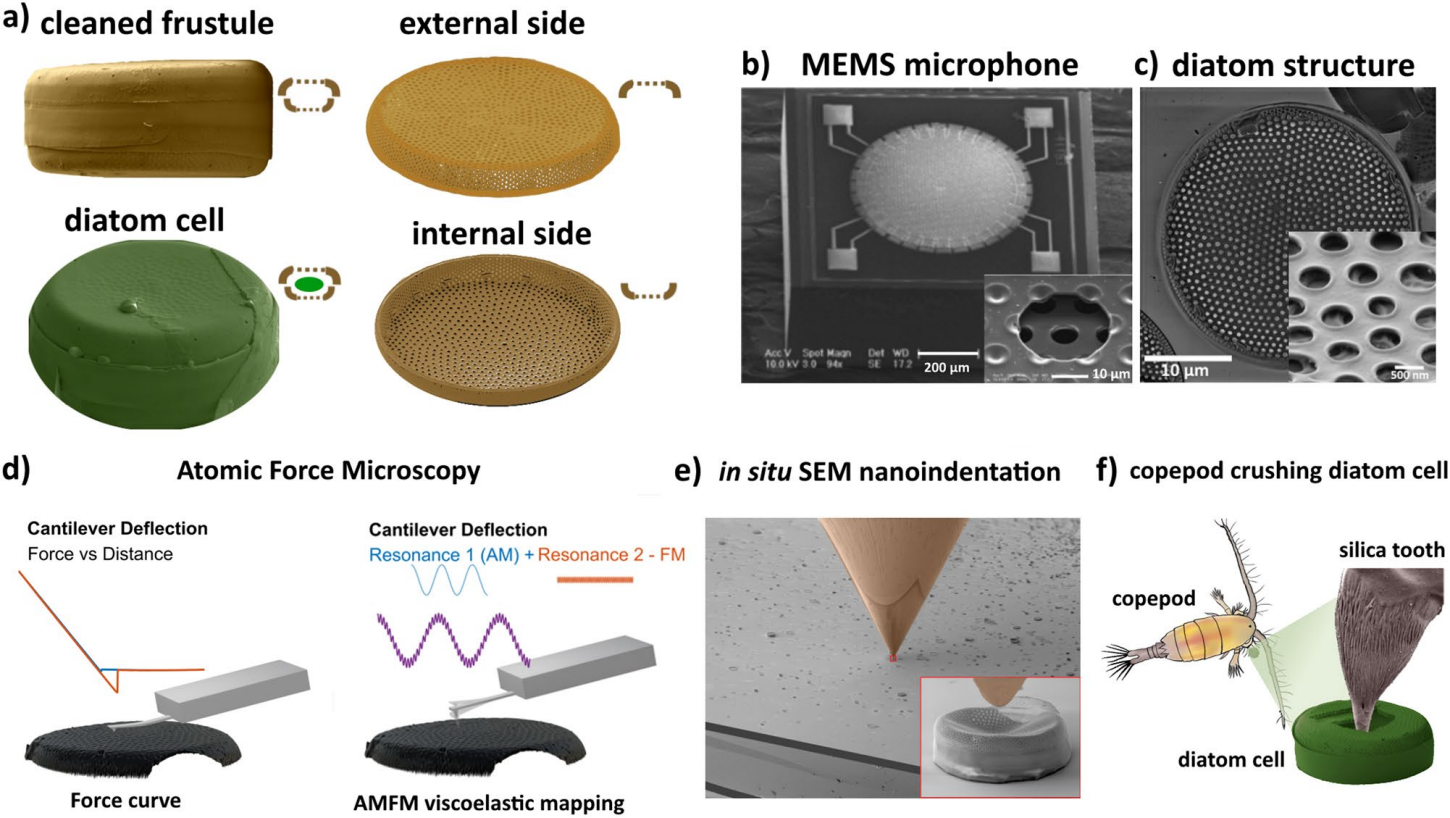
Study objects, biomimetic approach and research methodology. (**a**) Samples used in this study: cleaned frustule (organic matter removed), external and internal sides of cleaned frustules, diatom cell containing organic components, (**b**) SEM images of the MEMS microphone and the membrane perforation (inset). Adapted with a permission from^12^. (**c**) SEM image of diatom frustule showing the porous structure (inset), (**d**) Schematic diagrams of two modes of AFM measurement: force vs distance and amplitude modulation-frequency modulation (AMFM), (**e**) In situ nanoindentation inside SEM column, (**f**) Schematic illustration of a copepod crushing diatom cell with the help of a silica tooth as an example of indentation in nature.

Diatoms are of planetary ecological importance, making a huge contribution to biomineralization and carbon dioxide capture and oxygen production via photosynthesis. According to various estimates, diatoms form up to a quarter of the planet's organic material and release up to 20% of atmospheric oxygen^1–3^. The peculiar and distinguishing feature of diatom cells is the presence of a silica exoskeleton called frustule, a complex structure that ensures the performance of many vital functions of the unicellular micro-organisms—the interaction and exchange between diatom cells and the surrounding environment, protection from external mechanical influences, attachment to substrates, filtration, regulation of metabolism processes, control of light absorption during photosynthesis and the protection of DNA from UV radiation^4–7^. In terms of the number of species, pennate (bilaterally symmetric) diatoms clearly predominate over the centric diatoms accounting for only 19% of the nearly 17,000 diatom species within the accepted taxonomic description^8^. However, planktonic species, mainly centric ones, are responsible for the bulk of diatom population by numbers and volume, especially in nutrient-rich and well illuminated marine waters^9^. To proliferate in the shallow well-illuminated waters in the presence of predators they must protect themselves from various mechanical influences using a stable and lightweight structure. The requirements of small size, specific optical properties, low density and high strength are important for numerous applications, especially in silicon photonics^10,11^ and for microelectromechanical systems (MEMS)^12,13^. The interest in diatom frustules as prototypical photonic crystals for microelectronic technology arose relatively recently and has already been reflected in a number of high profile publications^14–20^. Dimensional (e.g. membrane thickness—1 µm, ratio between membrane holes^12^) and structural correspondence between diatom exoskeletons and devices such as photonic integrated circuits (PICs) and MEMS for high-sensitivity microphones (Fig. 1b) serve as a starting point for seeking further opportunities to create nanotechnology components using biomimetics and biotechnology^21^. Westerveld et al. fabricated two optomechanical ultrasound sensor in silicon photonics with membrane diameters of 20 and 15 μm consisting of the silicon dioxide lower cladding (BOX) and upper cladding (membrane)^10^. To obtain a mechanically stable membrane, they developed a PECVD (plasma-enhanced chemical vapour deposition) process for a 2-μm-thick mechanical SiO_2_ layer with low tensile stress.

To underpin the development of theoretical concepts in this area, comprehensive studies are required to include structural and mechanical characterization which remain small in number and limited to specific diatom species. Consequently, studies devoted to the possible use of diatom frustules as a basis for MEMS elements^22,23^ rely on unproven hypotheses or numerical simulation results^24^ that need validation.

The particular spatial organization of nano- and micropores of the diatom frustules offers promising prototypes of functional systems and elements for targeted drug delivery^25,26^, electrochemical energy storage^27,28^, photovoltaics^29^, metamaterials^30^, catalysis^31,32^, filtration^33^, metal modification and removal^20,34^ and many others^35–37^. The material, shape and thickness of the frustules, as well as the spatial order of the pores and the topology of the channels connecting them determine such engineering properties as stiffness and strength and natural oscillation frequency. However, the fine structure of the frustule porosity and the nanomechanical properties associated with it present challenges for the experimental study involving the application and detection of forces at scales relevant to the diatom cell. The elastic modulus, hardness, and tensile strength of various diatom species were studied using various experimental methods such as nanoindentation^38,39^, AFM probing^40–42^, and bending with glass microneedles^43^. In the most significant pioneering work of Hamm et al.^43^ the mechanical performance of the diatom frustule was assessed by performing loading tests with calibrated glass microneedles which led to the conclusion that the frustules evolved to provide necessary mechanical protection for the cells against predators. The studies performed by AFM and nanoindentation demonstrated significant variations of the elastic modulus depending on the location^38,40,42^ which may be due to the porous structure, nonuniform distribution of pores having different sizes and shapes, orientation of the frustule, location of the indent, etc. As the authors suggested^42^, improved instrumentation and the study of several species under physiological conditions are needed to gain more knowledge about the native mechanical properties and their link to morphogenesis, i.e., the process of silica biomineralization. To the best of our knowledge, there has been no report of a fundamental and systematic study of the relationship between the structural and topographic features of diatom shells as well as wet and air-dried cells containing organic material, and their mechanical characteristics under static and dynamic loading. Given the fact that the diatom frustule itself can be considered as a nanostructured membrane, the determination of the mechanical properties of both membranes and cells with all organic components present seems extremely important and necessary both from the point of view of fundamental research and from the point of view of the use of these systems as functional materials for new devices^44^. Inspired by nature, the combination of organic and inorganic components fits well with modern technologies such as Layer-by-layer assembly that can be readily applied to modify the inorganic substrate to fabricate multilayer composites^45^. Therefore, the purpose of this work is to establish relationships between a multilevel hierarchical structure and the behavior of materials with mechanical properties at the nano- and microlevels under different conditions that has direct effect on vibrational and acoustic properties of a frustule as ready complex mechanical system. It is our belief that a number of biomimetic and biotechnological approaches open a way to handle single frustules to ultimately apply them as a MEMS integrated to various sensor devices and other highly efficient engineering structures, the significance of which is undeniable.

## Results and discussion

### The biomimetic approach and research methodology

For the present study the centric diatom *Coscinodiscus oculus-iridis* was chosen as a representative sample with the most interesting variations in structural elements that can affect their mechanical and photonic properties. We investigated cleaned frustules consisting of two halves with different outer and inner surfaces, as well as wet and dried diatom cells containing organic components (Fig. 1a). The state of the sample interrogated is indicated by the small cartoons shown in the upper right corner to aid understanding. The striking similarity between diatom valves and the membranes used in modern MEMS microphones^12^ (Fig. 1b) is shown in Fig. 1c. Two different AFM modes used in this study are schematically illustrated in Fig. 1d, whilst in situ nanoindentation in the SEM column is shown in Fig. 1e. The details of these experimental approaches are explained in the Materials and Methods section. The mechanical contact between a sharp indenter and diatom frustule is a frequent occurrence in nature in the course of interaction with marine zooplankton. For example, several species of copepods use the force of their mandibles to crush diatoms using silica-tipped parts called opal teeth^46^, as illustrated in Fig. 1f.

### Morphology and topography study: from micro to nanoscale

**Figure 2:**
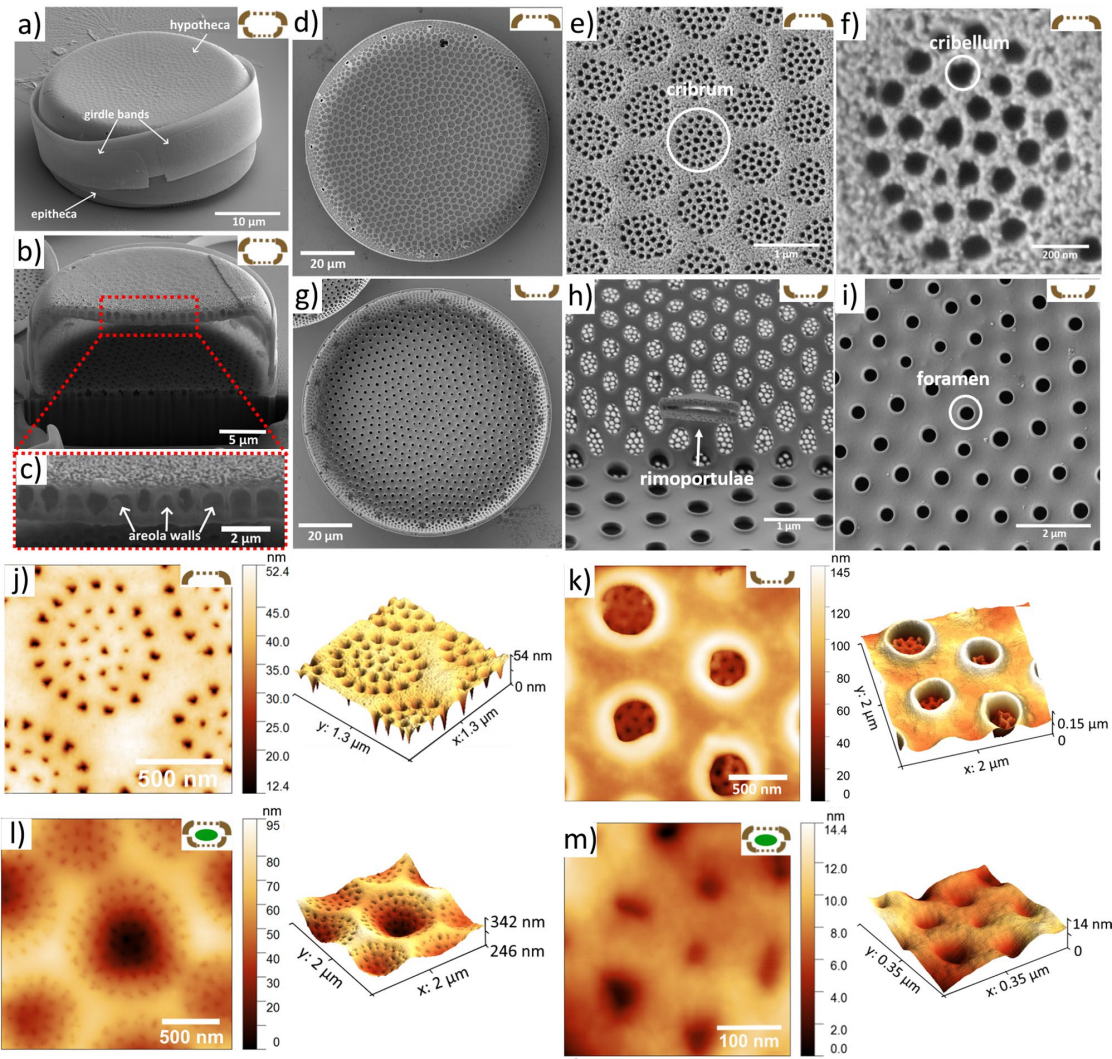
Microscopic images of diatom frustule. SEM images of: (**a**) the cleaned diatom frustule consisting of a hypotheca and an epitheca joined together by girdle bands, (**b**) the frustule FIB cross-section showing areola walls (**c**, **d**) outer surface of the valve, (**e**) details of the external side of the valve: cribrum, (**f**) cribellum, (**g**) inner surface of the valve, (**h**) details of the internal side of the valve: rimoportulae, (**i**) foramen. AFM topography of: (**j**) an outer surface of the cleaned frustule decorated with porous domes (cribrum), (**k**) foramen holes on the inner surface of a cleaned frustule, (**l**) a surface of a wet diatom cell forming a hilly terrain, (**m**) close view of regularly spaced pores on the outer surface (cribellum) of a wet diatom cell.

Figure 2 shows morphology and topography of *Coscinodiscus oculus-iridis* first identified by Ehrenberg in 1839.

The frustule shown in Fig. 2a consists of two valves: the upper epitheca and the lower hypotheca, connected by a series of siliceous rings called girdle bands^47^. The diameter of valves ranges from 30 to 70 µm, while their height varies from 10 to 15 µm, although much larger size ranges can be found in literature. The cross-sectional view of a diatom frustule after FIB (Fig. 2b,c) demonstrates the multi-layered hierarchical structure of the cell wall, formed by orthosilicic acid in silica deposition vesicle^36,48^. The wall thickness is in the 0.3–1.5 µm range. The disk-shaped valve faces are slightly depressed in the central area, which is frequently less silicified than the peripheral region^49^. The outer thin perforated silica layer called cribrum (Fig. 2d,e,j) consists of a regularly spaced array of 40–70 nm pores (cribellum)^50^ (Fig. 2f,m). The pore-to-pore distance in one array is around 100 nm. The honeycomb-like chambers called areolae are open to the cell interior via rimmed circular openings (Fig. 2g)—foramen with a diameter of around 0.2–0.5 µm (Fig. 2h,i). The porous pattern of the cribrum layer is located exactly above the foramen. The rimoportula openings having the shape of a pair of lips are distributed along the perimeter of the internal valve (Fig. 2h). On the external valve face, the rimoportula opening is a simple, round aperture, through which they extrude polysaccharides and other carbon compounds. AFM topography images (Fig. 2j–m) show that the surface of the air-dried live diatom cell containing organic material is not flat and monotonously "smoothed", but form a hilly terrain (Fig. 2l,m). Organic components of diatoms can be classified in the following way: (1) an organic casing represented by a thin layer surrounding silica wall, (2) the diatotepum or diatotepic layer located between the plasmalemma and the silica, (3) molecules or organic complexes trapped within the silica, (4) mucilage associated with the cell surface or secreted by diatoms which can be used for motility, adhesion or protection^51^. On the cleaned frustule the perforations on the outermost surface are simple openings (Fig. 2j), while on the inner surface they have thickened rims (Fig. 2k).

The analogy between the natural appearance of silica diatom frustule lase structure and artificial MEMS (especially microphone membrane) served as a motivation to carefully study diatoms as a mechanical system. Mechanical sensitivity of the MEMS capacitive microphones increases when the thickness of the diaphragm increases^52^. However, it is important to find an optimal size correlation between thickness and mechanical response. On the one hand, a thin membrane with a large radius has more flexibility, but at the same time can be brittle. On the other hand, a thick membrane with a small radius has a small flexibility and, therefore, poor flexibility. According to the review article^53^, the average membrane diameter is 600 µm, while the thickness makes up 1 µm, which corresponds well to the silica frustule thickness.

### AFM and AMFM studies of mechanical properties of cleaned frustules and wet diatom cells in static mode

**Figure 3:**
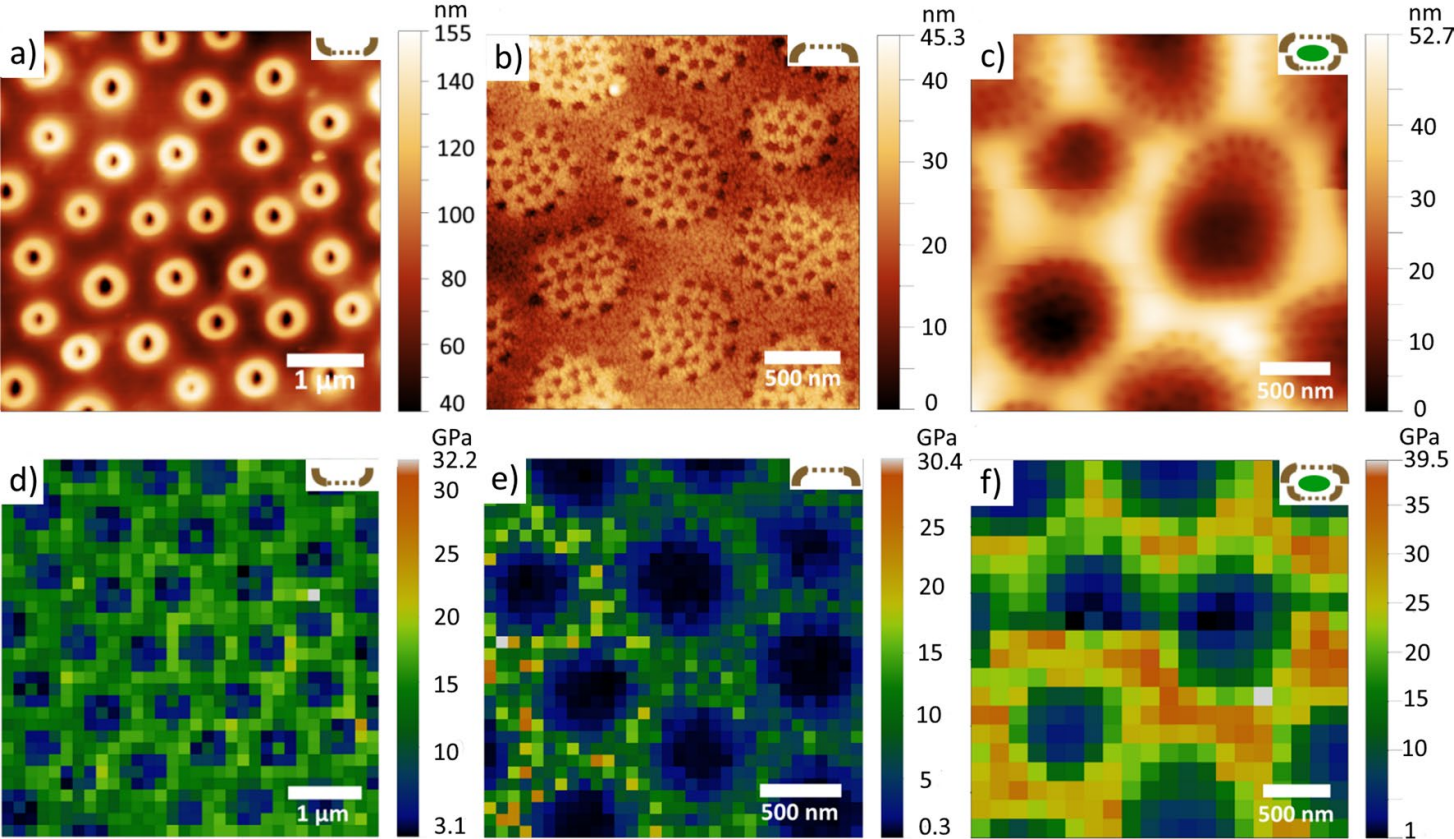
AFM study of cleaned frustules and wet diatom cells. Topography of a measured: (**a**) inner side and (**b**) outer side of cleaned frustule, (**c**) wet diatom cell. Young’s modulus of: (**d**) inner and (**e**) outer surface of cleaned frustule, (**f**) wet diatom cell.

Figure 3 shows AFM topography and corresponding maps of Young’s modulus measured on the inner (Fig. 3a,d) and the outer (Fig. 3b,e) surfaces of cleaned diatom frustules and on the wet cell containing organic material (Fig. 3c,f). The Young’s modulus data were obtained by collecting force-distance curves in the elastic regime.

The results show that Young’s modulus of the thick space between pores is E = 15 ± 2 GPa and E = 10 ± 4 GPa for the inner and the outer surfaces of cleaned frustules, respectively, while for the wet diatom cell E = 25 ± 5 GPa, which was expected considering the support from the organic material trapped inside the frustule. According to study^54^, the introduction of molecular water into the silica network leads to an increase of Young's modulus of silica glass at low water content. On the other hand, due to excessive boiling in concentrated nitric acid, the frustule material can become friable and fine structures in some cases may be lost which leads to the lower mechanical performance. Conversely, if the oxidation was weak and the samples were poorly washed, the organic matter lays down on the surface of the frustule in a thin layer, covering and masking fine structures. The stronger mechanical structure of the internal plate is expected as this layer is the basic framework for building the other porous layers—cribrum and cribellum. Note, however, that the values fall within the error margins (Supplementary Fig. 1). Finite element simulation of an isolated pleura of *T. punctigera*^43^ showed that the Young's modulus of diatom silica is 22.4 GPa, which is comparable to cortical bone (20 GPa) and in good agreement with our study. Young’s modulus of the rims around pores is lower probably because the material of the rims is not clamped in in-plane direction, as shown in Supplementary Fig. 2.

**Figure 4:**
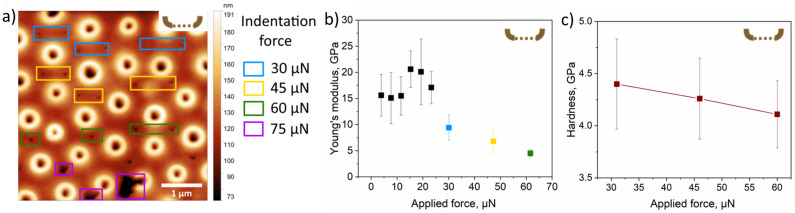
AFM indentation at specific points of a cleaned inner valve. (**a**) Inner surface of a frustule measured by the stiff probe after indentation at specific points by applying different indentation force, (**b**) Young’s modulus calculated using the Derjaguin–Muller–Toropov (DMT) model at different applied forces after averaging over 10 points, (**c**) the dependence of hardness on the applied force.

We also performed indentation at specific points on the inner surface of the cleaned frustule by applying different indentation force, as indicated in Fig. 4a.

Young’s modulus calculated from these data using the DMT model (Fig. 4b) show the same trend as for the reference fused silica sample (Supplementary Fig. 1), i.e., correct values are at the applied force below 20 µN where the tip-surface contact geometry may be approximated by a sphere and interaction is mainly elastic. At higher forces the model does not fit well because the contact geometry deviates from the spherical one, while at 75 µN the valve breaks. Hardness calculated as a ratio of the maximum indentation force to the indent area is between 4.0 and 4.5 GPa and slightly decreases with increasing loading force, which is typical for nanohardness measurements (Fig. 4c)^55^. Similar behavior was observed on the reference fused silica sample (Supplementary Fig. 1). The hardness of the measured cleaned inner valve was significantly higher as compared to the previously reported^40^ values obtained on the outer porous layers [(0.033–0.116 GPa)—at the center, (0.076–0.120 GPa)—at the edge]. The Young’s modulus values obtained in our study were higher than in the study by Losic et al.^40^, where they varied from 0.591 to 2.768 GPa at the center of the frustule and from 0.347 to 2.446 GPa closer to the edge.

**Figure 5:**
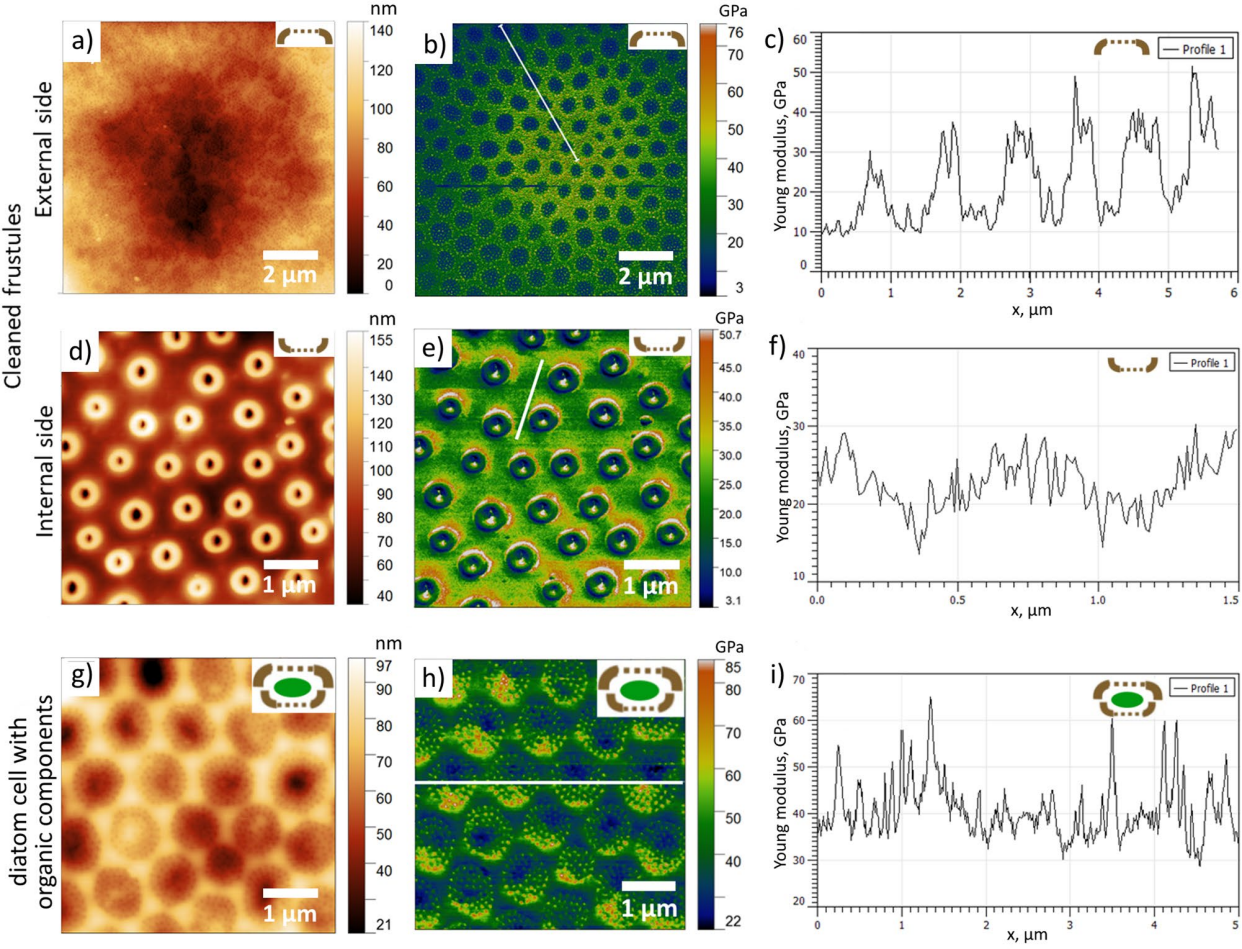
The AMFM study of cleaned frustules and wet diatom cells. External side of the cleaned valve: (**a**) Topography, (**b**) The AMFM Young modulus, (**c**) The AMFM Young modulus profile along the white line shown in (**b**). Internal side of the cleaned valve: (**d**) Topography, (**e**) The AMFM Young modulus, (**f**) The AMFM Young modulus profile along the white line shown in (**e**). External side of the wet diatom cell: (**g**) Topography, (**h**) The AMFM Young modulus, (**i**) The AMFM Young modulus profile along the white line shown in (**h**).

Young’s modulus on the cribrum in Fig. 3 appears smaller than on the thicker parts of the sample. It can be explained by the fact that the cribrum is so thin that during force-distance curve acquisition it bends as a membrane even at low applied force (100 nN). In this case the obtained values of Young’s modulus do not correctly reflect properties of the material. In order to obtain more accurate results for the cribrum, we used a gentler AMFM viscoelastic mapping method^56^, in which the AFM cantilever scans the sample’s surface in the tapping mode in the repulsive regime^57^ being excited simultaneously at two resonance frequencies. The results are shown in Fig. 5.

The AMFM Young’s modulus is up to two times higher than the modulus calculated from the force-distance curves. On the outer surface of the cleaned frustule Young’s modulus of the cribrum is about 3 times lower than of the thicker space in between, while on the wet diatom cell, where the inorganic frustule is supported by the organic interior, the difference almost vanishes. This result implies that Young’s modulus calculated on the cribrum from the force-distance curves is indeed affected by the membrane effect and is lower than the real one.

**Figure 6:**
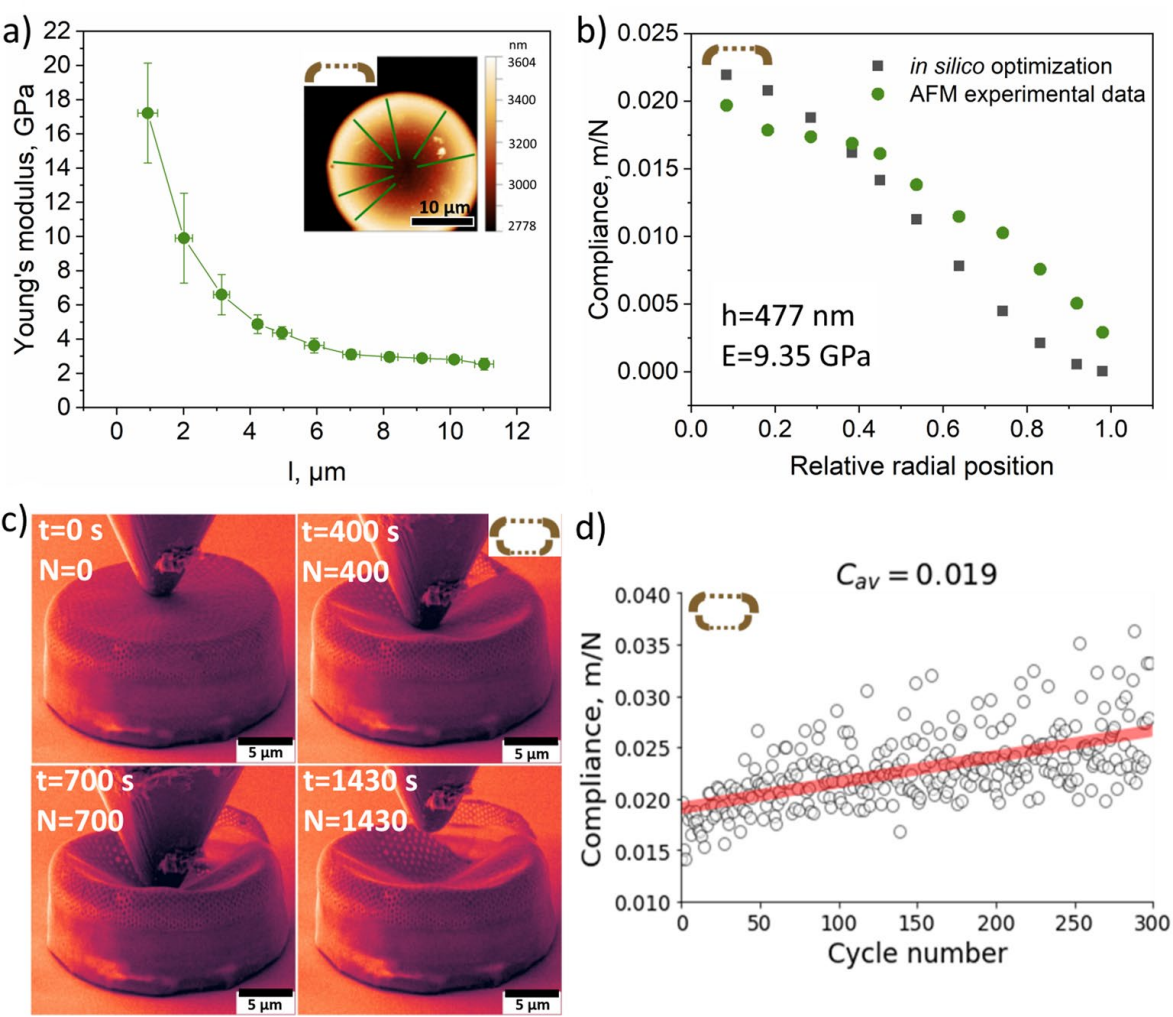
Compliance of cleaned frustules: static vs. dynamic approach. (**a**) Young’s modulus depending on the distance from the edge towards the center of the outer surface of the valve. Inset: topography of outer surface of a cleaned frustule. The measurements were carried out using AFM by pressing the diamond probe on the sample with a force of 7.7 µN along each green line. (**b**) Compliance vs. relative radial position calculated using the Green’s function of a point force, (**c**) SEM images of whole diatom frustule acquired during different stages of nanoindentation in the SEM column, (**d**) Calculated compliance vs. cycle number. Video material corresponding to Fig. 6c is provided in Supplementary Materials (see Supplementary Video 1).

To get deeper insight into mechanical properties of the cleaned valve we acquired force-distance curves from the edge towards the center of the outer surface along green lines shown in Fig. 6a using the stiff diamond probe. The maximum loading force of 7.7 µN falls into the elastic regime (without indentation) and the frustule’s outer surface bends as a membrane under the load. The results showed that the bending increases from the side towards the center of the valve. Young’s modulus calculated using the Hertz fitting model decreases as the distance from the edge towards the center of the valve increases.

There is a strong correlation between MEMS' performance and the mechanical properties of the materials they are constructed from. By selecting, designing, fabricating, and packaging materials in accordance with mechanical properties, some of the mechanical failure modes may be eliminated^58^. Polysilicon, the most frequently used MEMS material, has Young’s modulus mostly in the range between 160 and 180 GPa, similar to single-crystal silicon (160 – 190 GPa) ^58^. Silicon-carbide and silicon–nitride are also promising MEMS materials having Young’s modulus values determined by indentation method 395 GPa and 101 – 251 GPa, respectively^58^. Silicon oxide is often included in MEMS devices (e. g. as a sacrificial material in polysilicon surface micromachining), but has low stiffness and strength (64 GPa^58^).

## Compliance of cleaned frustules: Static vs. dynamic approach

The data obtained was used in combination with the analytical model for circular membrane deformation given by Melnikov^59^. The Green’s function of a point force $G(z, \zeta )$ for Kirchhoff plates satisfies the equation:

1 $$\begin{array}{c}D{\nabla }^{4}G\left(z,\zeta \right)=\delta \left(z-\zeta \right)\end{array}$$

where $D=E{h}^{3}/12\left(1-{\nu }^{2}\right)$ is the flexural rigidity of the diatom valve given in terms of Young’s modulus $E$, thickness $h$ and $\nu$ is Poisson’s ratio, while $z = r(\mathrm{cos}\varphi + i\mathrm{ sin}\varphi )$ and $\zeta = \rho (\mathrm{cos }\psi + i \mathrm{sin} \psi )$ represent the observation point and the force application point, respectively. The solution is given by:

2 $$\begin{array}{c}G\left(z,\zeta \right)=\frac{1}{8\pi D}\left[\frac{1}{2{a}^{2}}\left({a}^{2}-{\left|z\right|}^{2}\right)\left({a}^{2}-{\left|\zeta \right|}^{2}\right)-{\left|z-\zeta \right|}^{2}\mathrm{ln} \frac{\left|{a}^{2}-z\underset{\_}{\zeta }\right|}{a\left|z-\zeta \right|} \right]\end{array}$$

where *a* is the radius of the diatom. By fitting the model to experimental observations, the membrane flexural rigidity can be used to determine the overall apparent Young’s modulus of the diatom valve as a circular membrane. The compliance defined as the measure of the structure deformation under the action of external forces was calculated as the reciprocal of rigidity. The dependence of compliance on the relative radial position calculated using influence function of a point force is shown in Fig. 6b. Maximum similarity with the experimental values was achieved at *h* = 477 nm and *E* = 9.35 GPa. In the study by Shubham et al.^13^, the mechanical compliance of a semiconstrained polysilicon diaphragm with peripheral and center protrusions on the backplate is calculated to be 7.154 × 10^−3^ m/N, which is in good correspondence with our results, as demonstrated in Fig. 6b.

The results of cyclic loading (amplitude 1 µm, period 1 s) performed on cleaned diatom frustule in the SEM column are shown in Fig. 6c, Supplementary Fig. 3, and Supplementary Videos 1, 2. Based on the dependence of the displacement on time, considering sinusoidal motion represented as A·sin(ωt), we found the stiffness and subsequently compliance as a function of cycle number, as presented in Fig. 6d. Compliance increases with increasing number of cycles. The average compliance was found to be 0.019 m/N. The frustule begins to break along one edge after ca. 300 s, but continues to oscillate during cyclic loading without complete rupture.

**Figure 7:**
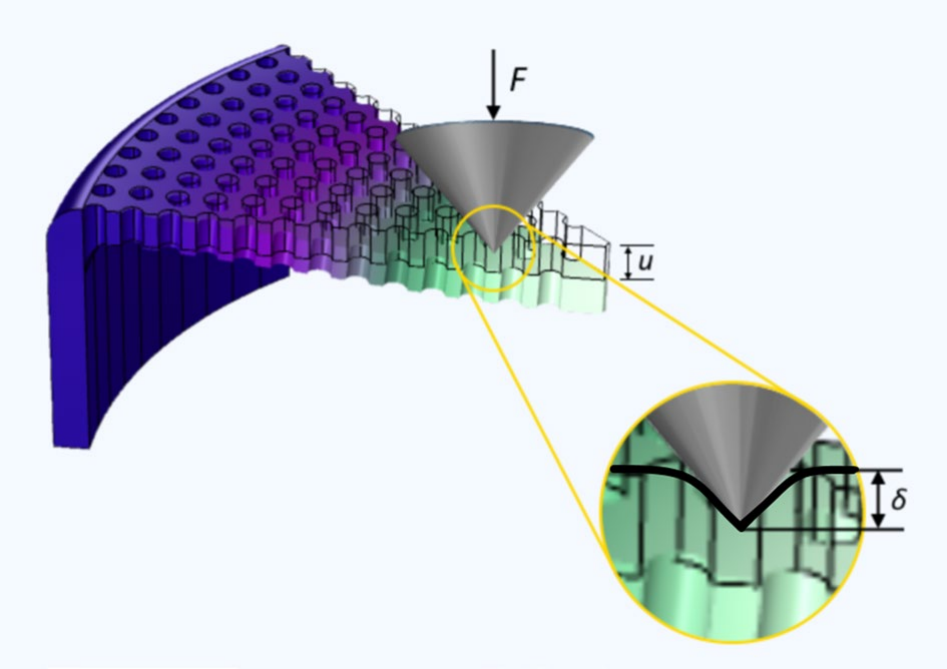
The deformation response of the frustule valve to local indentation.

Considering the deformation response of the frustule valve to local indentation illustrated in Fig. 7, the total compliance of the system can be written as the sum of the local material compliance under the indenter, ${C}_{L}$, and the global structural compliance ${C}_{G}$:

3 $$\begin{array}{c}C=\frac{\delta +u}{F}=\frac{\delta }{F}+\frac{u}{F}={C}_{L}+{C}_{G}\end{array}$$

It is well known from the literature for elastic^60,61^ as well as plastic indentation^62^ that the force–displacement relation is described by a power law function of the type $F\cong {\delta }^{(1+m)}$, where $m>0$. Therefore, the local compliance decays with increasing applied force as ${C}_{L}\cong {F}^{-m}$, whilst the global compliance ${C}_{G}$ remains appreciably constant within the range of forces for which the structural deflection remains elastic. By the same argument, the local compliance ${C}_{L}$ dominates over global compliance ${C}_{G}$ for small applied forces. This transition between the two regimes takes place over a range of loads that depend on the material properties and the indenter shape. It can therefore be concluded that both the local material and global structural responses can be interrogated via the suitable choice of loading conditions and indenter tips under the small and large applied forces, respectively. Both local and global effects can be considered together only once the global compliance has been calibrated.

### Mechanical response from cleaned frustules vs. dried diatom cells measured by in situ SEM nanoindentation

**Figure 8:**
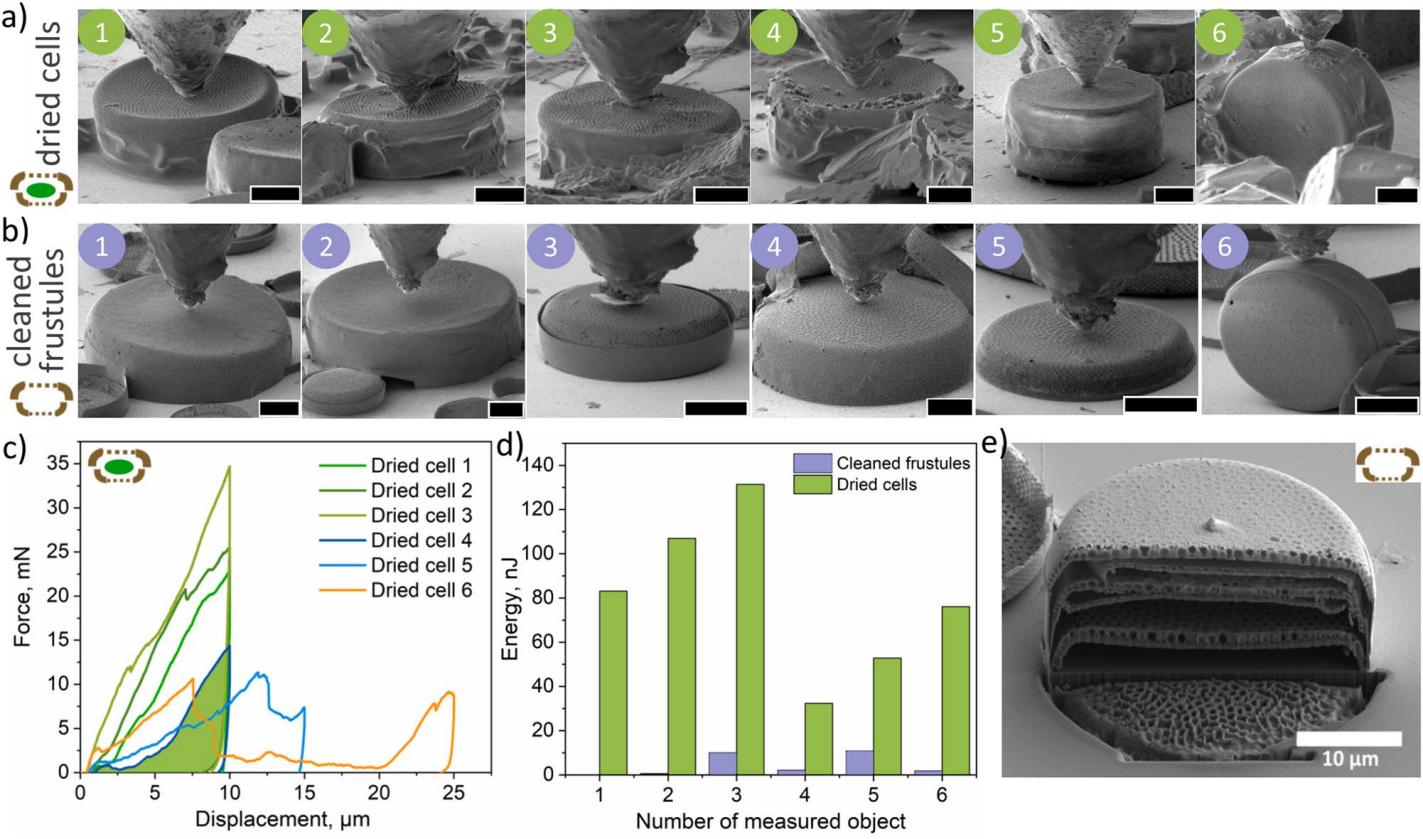
In situ SEM nanoindentation of cleaned frustules and dried diatom cells. (**a**) SEM images of air-dried diatom cells measured by nanoindentation in the SEM column, (**b**) SEM images of cleaned frustules. Scale bar, 10 µm. (**c**) Force–displacement curves obtained during indentation of dried cells, (**d**) Calculated area under the force–displacement curves of dried and cleaned diatoms equal to work done, (**e**) The case of a layered structure of the frustules of some diatoms, potentially responsible for difference in the obtained force-distance curves. Video material corresponding to (**a**, **b**) is provided in Supplementary Materials (see Supplementary Videos 3–13).

We also performed static in situ nanoindentation inside SEM on dried cells with cellular material (Fig. 8a) as well as cleaned diatom frustules (Fig. 8b) and based on the force- displacement curves we analysed their mechanical performance. Static studies lead us to a more precise definition of the module of a material as a whole. Figure 8c shows the force–displacement curves of dried cells, which vary depending on the orientation and size. As can be seen, dried cells 1–4 (diameter, 37–40 µm; height, 11–13 µm) have a similar size and mechanical behavior, which is different from the dried cell 5, whose diameter/height ratio is smaller (diameter, 41 µm; height, 18 µm). On the other hand, we observed a difference in mechanical performance when indenting a dried cell 6 from the girdle band point.

The nature of the curves of cleaned frustules and dried cell with organic material is different (Supplementary Fig. 4). Also, the forces are significantly lower in the case of cleaned frustules, as well as the area under the curves, which is equal to work done on the object (Fig. 8d). With the help of AFM, we made local indentations which gives us more information about material, however we did not completely deform the structure, as in the case of nanoindentation in SEM column performed with the blunt tip indenter. The differences between the values can also be attributed to the layered “burger” structure within the frustule of some diatoms, as demonstrated in Fig. 8e. This situation can occur in old cultures when the process of cell division is corrupted and formation of the valves is not accompanied by cytokinesis. The combination of organic and inorganic components in diatom cells, leading to their superior mechanical characteristics, can serve as an inspiration for layer-by-layer assembly, which can be used to modify the inorganic substrate for the manufacture of multilayer composites^45^.

## Conclusions

Here, we discuss the mechanical performance of diatoms in the pursuit of the idea that there is a close relationship between the structural design of diatom frustules, the diversity of their appearance and various parameters, including porosity, location of the indentation, orientation, organic material support, and silica hydration. We applied AFM and in situ SEM nanoindentation methods to study *Coscinodiscus oculus-iridis* cleaned frustules and cells containing organic material at the micro and nanoscale under static and dynamic loading. The Young’s modulus of wet diatom cells was significantly higher (E = 25 ± 5 GPa) compared to cleaned frustules (E = 15 ± 2 GPa (inner surface) and E = 10 ± 4 GPa (outer surface)). The AMFM viscoelastic mapping method gave up to two times higher Young’s modulus values of the measured samples compared to the values calculated from the force-distance curves. Besides static in situ nanoindentation measurements, we demonstrated for the first time the behavior of the frustule under the cyclic loading, which is vital for the application of diatom-like structures in MEMS devices. The nanoindentation measurements in the dynamic mode showed that the average compliance of a cleaned frustule is 0.019 m/N. We also discussed the total compliance as the sum of the local material compliance under the indenter and the global structural compliance. We believe that diatoms offer a wide selection of attributes for successfully solving the fundamental problems of bionics, namely the effective application of the principles of building their exoskeleton to create highly efficient microelectromechanical and photonic devices.

## Materials and methods

### Diatom cultivation

*Coscinodiscus oculus-iridis* (Ehrenberg) Ehrenberg 1840 was isolated from samples collected at a depth of 2 m in the Tsushima Strait in 2020. The sample was delivered to T.I. Vyazemsky Karadag Scientific Station. Monocultures were isolated in the form of single cells using Pasteur micropipettes, which in the process of division formed clonal cultures. Clone 20.1211-OD was used in the present work. Cultures were grown in ESAW medium prepared according to a modified recipe as described by Polykova et al.^63^ with salinity of ca. 36‰. Erlenmeyer flasks with diatom cultures (ca. 50 ml) were kept on a north-facing windowsill of the laboratory at room temperature under natural light. To maintain the exponential growth phase, the cultures were re-inoculated to a new medium once a week.

### Preparation of samples for SEM and AFM

Cleaned diatom frustules and air-dried living cells were prepared for AFM and in situ SEM nanoindentation measurements. Purification of diatoms from organic material consists of several stages. For the purpose of bacterial destruction of organic matter, the medium was first filled with distilled water and left for 3 days. After that it was washed with DI water several times to remove the remaining salts by centrifugation using the Centrifuge 5340 (Eppendorf, Germany) (500×*g*, 5 min). After removing salt, the cell suspension was boiled in concentrated nitric acid for 2–3 h. The boiling process was repeated the next day adding fresh nitric acid. The suspension was subsequently centrifuged (500×*g*, 5 min) and rinsed with DI water at least 8 times. The cleaned frustule suspension was pipetted onto a silicon wafer and air-dried for further measurements. Air-dried living diatom cells containing cellular material were also placed on a silicon wafer and used for in situ SEM nanoindentation. A vial with live diatoms in water was transferred into the AFM glovebox. A drop of water with diatoms was placed on a silicon substrate. After water evaporation the live diatom cells were measured using the AFM. The diatom samples were imaged with intact and separated frustules or the so-called valves. Separated frustules settled on the substrate exposing their interior concave surface and exterior convex surface.

### Scanning electron microscopy (SEM)

Scanning electron microscopy images were acquired using a TESCAN CLARA (Brno, Czech Republic) electron microscope. Samples were mounted on a standard aluminum stub using carbon adhesive tape and visualized without sputter coating in a high vacuum at 1 keV landing energy and 30 pA beam current. The cross-sections of cleaned diatom samples were obtained by focused ion beam (FIB) under high vacuum in a TESCAN SOLARIS S9000 (TESCAN ORSAY HOLDING, Brno, Czech Republic) electron microscope.

### Atomic force microscopy (AFM)

AFM measurements were performed using Cypher ES microscope (Asylum Research, Oxford Instruments) installed inside an Ar filled glove box (MBraun). Topography was measured in a tapping mode using a single crystal diamond probe with 147 kHz first resonance frequency and 3.5 N/m spring constant. The nanomechanical properties were measured in the AM-FM viscoelastic mapping mode^55^ and by force-distance curve acquisition^56^ using stiff (DRP_IN purchased from TipsNano, Estonia, k = 400 N/m, F_res_ = 750 kHz) and soft (HA_NC/FD purchased from TipsNano, Estonia, k = 3.5 N/m, F_res_ = 147 kHz) single crystal diamond probes. Nanoindentation was performed on the concave surface of a separated frustule using the stiff (k = 400 N/m) diamond probe. All AFM probes were calibrated on a TGT1 test grating in order to measure radius of curvature of the tip apex and on a fused silica reference sample in order to test models for fitting experimental data. The stiff probe was additionally calibrated on a diamond coated silicon wafer in order to get accurate values of the displacement and spring constant. The DMT model was implemented for data fitting and analysis. Detailed description of the fitting model is given in Electronic Supplementary Information. Force-distance curves on the inner surface of the cleaned frustule and on the wet diatom cell were acquired by the stiff diamond probe (k = 400 N/m) under the maximum force load of 8 µN. Force-distance curves on the outer surface of the cleaned frustule were obtained by the soft diamond probe (k = 3.5 N/m) under the maximum force load of 100 nN because the cleaned cribrum is brittle and often breaks when measured by the stiff probe. Besides, the outer surface bends as a membrane under the stiff probe. Image processing and analysis was performed using Gwyddion software.

### In situ SEM nanoindentation

The Alemnis Standard Assembly (ASA) indentation platform (Alemnis, Gwatt, Switzerland) was used to perform in situ nanoindentation testing of cleaned frustules and air-dried living diatom cells placed on silicon wafer inside the chamber of a TESCAN SOLARIS S9000 SEM (TESCAN ORSAY HOLDING, Brno, Czech Republic). In the current work, the ASA tester was equipped with a flatpunch conductive diamond indenter with the diameter of 2.5 μm on the cutting edge and 60° cone angle. The experiment implied synchronization of two parallel processes like SEM images acquisition and indentation test. In order to achieve the best SEM resolution considering design limitations of the devices, ASA platform was tilted on 20° and moved to the working distance of ~ 7 mm. The SEM images were recorded under the following conditions: high voltage of 5 kV, beam current of 1 nA, spot size of 15 nm, pixel size of 40 nm and recording speed of 0.5 fps. The nanoindentation testing was done in both static and dynamic modes. The static mode was done under the regime of the linear depth control where the test consisted of three stages as indenter approach, holding and indenter retraction. The dynamic mode included a sinusoidal signal instead of a holding stage. The penetration depth was estimated for each diatom cell height before the test. Videos of nanoindentation process of both types of objects can be found in the Supplementary Information.

## Supplementary Information

Supplementary Information 1.

Supplementary Video 1.

Supplementary Video 2.

Supplementary Video 3.

Supplementary Video 4.

Supplementary Video 5.

Supplementary Video 6.

Supplementary Video 7.

Supplementary Video 8.

Supplementary Video 9.

Supplementary Video 10.

Supplementary Video 11.

Supplementary Video 12.

Supplementary Video 13.

## Data Availability

All data generated during this study are included in this article.
