# Supplementary material for: Revealing the static and dynamic nanomechanical properties of diatom frustules—Nature's glass lace
Source: Sci Rep. 2023 Apr 4;13:5518. doi: 10.1038/s41598-023-31487-x (PMC10073200; doi:10.1038/s41598-023-31487-x)
Supplement: Supplementary file 1 — Supplementary Information 1. [file 41598_2023_31487_MOESM1_ESM.docx]

Electronic Supplementary Information for

**The nanomechanics of diatom silica frustules, Nature’s glass lace**

Julijana Cvjetinovic^1,*^, Sergey Yu. Luchkin^2^, Eugene S. Statnik^2^, Nickolai A. Davidovich^3^, Pavel A. Somov^2^, Aleksey I. Salimon^2,4^, Alexander M. Korsunsky^5^, Dmitry A. Gorin^1,*^


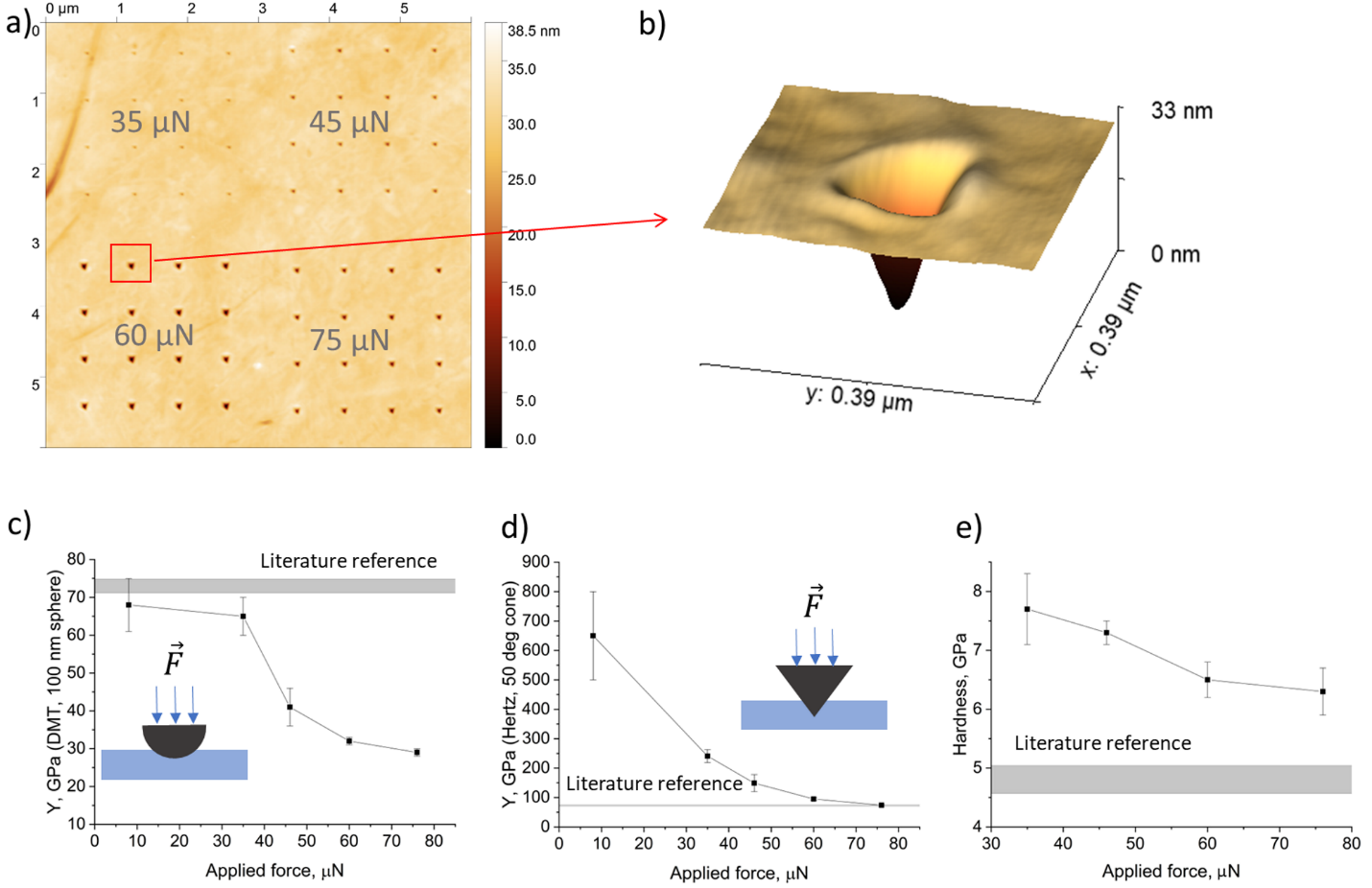


**Supplementary Fig. 1**. **Calibration of a stiff diamond probe (k=400 N/m) on a fused silica reference sample.** **a** Topography, **b** The stiff diamond cantilever has a spherical tip apex with R ≈ 100 nm and a pyramid body with half angle ≈ 45 deg. During indentation the contact geometry changes from spherical at low force to pyramidal at high force. Different models were used to fit force-distance curves and calculate Young’s modulus. **c** The DMT model works fine (in agreement with literature reference) at low indentation force (< 30 µN), where the tip-sample contact geometry is mainly determined by the tip apex (assuming flat surface). **d** The Hertz model works fine (in agreement with literature reference) at high indentation force (> 70 µN), where the tip-sample contact geometry is mainly determined by the tip body (assuming flat surface). **e** Hardness was calculated as a ratio of indentation force to the indent area. The measured hardness is higher than the literature reference, but of the same order of magnitude. The difference might be due to the size effect, since the AFM indents are much smaller than the Vickers indents.

Different models were used to fit force-distance curves and calculate Young’s modulus. The stiff diamond probe has a spherical tip apex with R ≈ 100 nm and a pyramidal body with half angle ≈ 45 deg. During indentation the contact geometry changes from spherical at low indentation force to pyramidal at high force as the tip indents the sample. Using the reference fused silica sample (Supplementary Fig. 1) we established that at the low indentation force (≤20 µN) where the tip-sample contact geometry is mainly determined by the tip apex (assuming flat surface) the DMT (Derjaguin-Muller-Toropov) model gives results in agreement with literature reference, which can be found elsewhere. Here the DMT model assumes a spherical indent with R=100 nm made of synthetic diamond, and the sample’s Poisson ratio 0.15. At the high indentation force (>70 µN) where the tip-sample contact geometry is mainly determined by the tip body, the Hertz model gives adequate results. The Hertz model assumes a conical indent with 50° half angle made of synthetic diamond, and the sample’s Poisson ratio 0.15. For the diatom samples we applied low loading force and implemented the DMT model except for the hardness measurements.


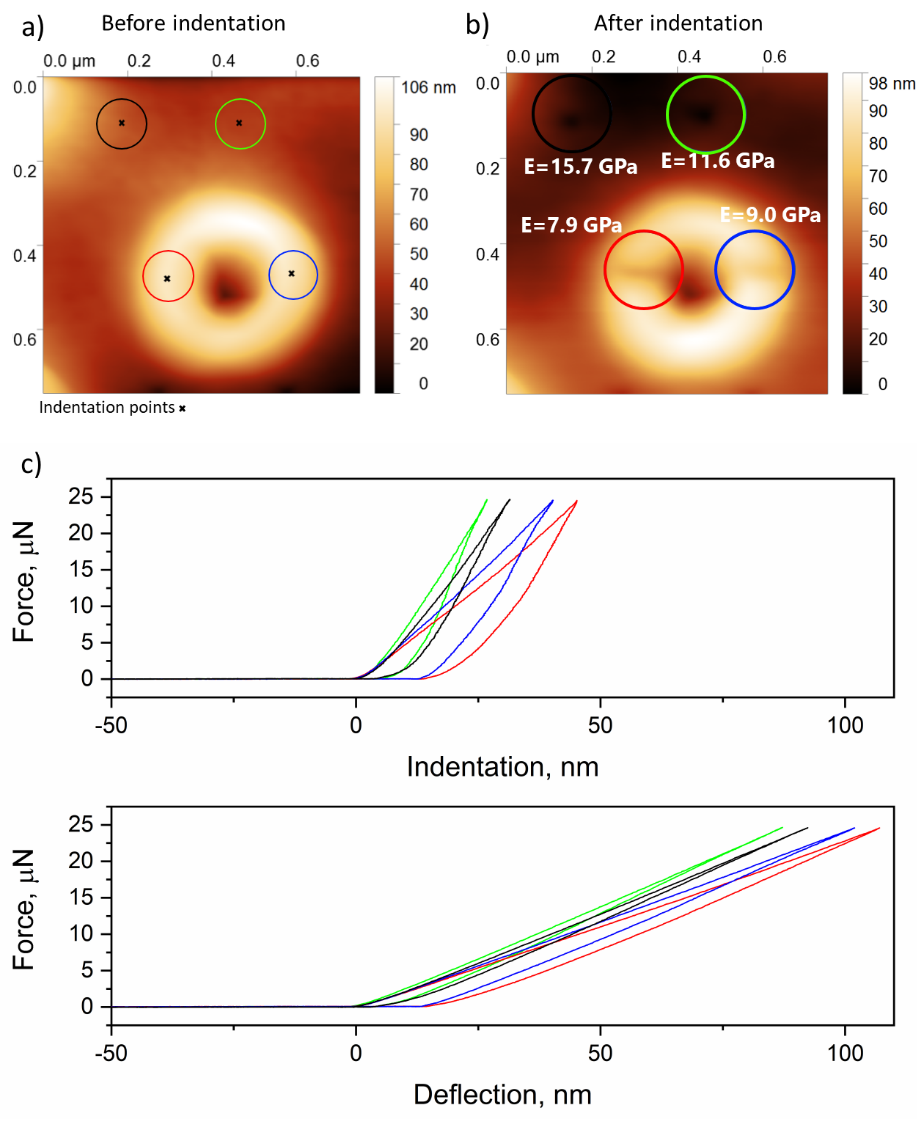


**Supplementary Fig. 2.** **Inner surface of a frustule measured by the stiff diamond probe (k=400 N/m).** **a** Topography before nanoindentation, **b** topography after nanoindentation with indicated Young’s modulus values calculated using DMT model, **c** force-indentation and force-deflection curves collected at specific indentation points.


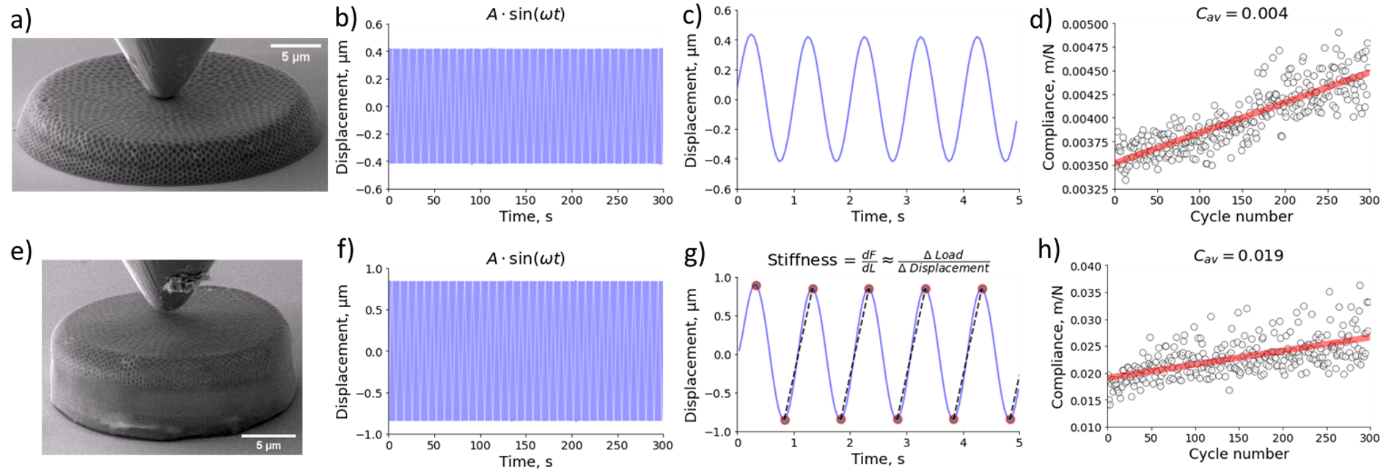


**Supplementary Fig. 3.** **Nanoindentation in the SEM column – cyclic loading**. **a** SEM image of a measured cleaned diatom upper valve, **b** Displacement vs. time, the whole region – 300 seconds, **c** Displacement vs. time, a short time interval – 5 seconds, **d** Compliance vs. cycle number, **e** SEM image of a measured cleaned diatom frustule, **f** Displacement vs. time, the whole region – 300 seconds, **g** Displacement vs. time, a short time interval – 5 seconds, **h** Compliance vs. cycle number. Amplitude 1 µm, period 1s.


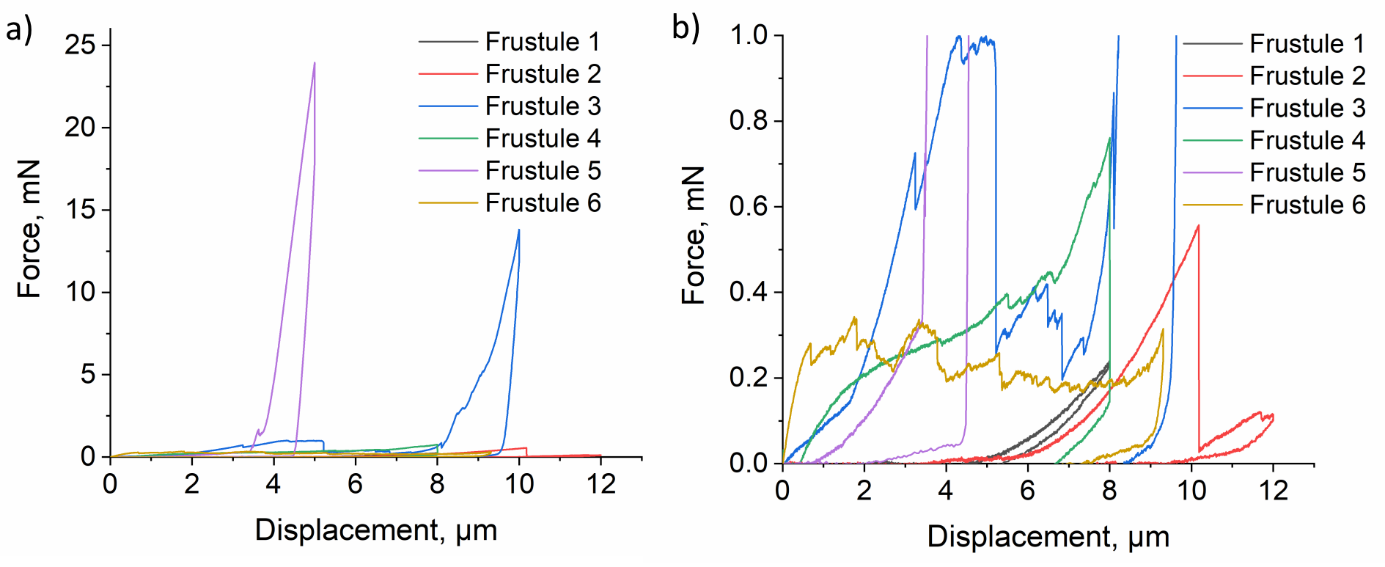


**Supplementary Fig. 4.** **Force-displacement curves of cleaned frustules obtained by in situ SEM nanoindentation.** **a** force-displacement curves for forces up to 25 mN, **b** magnified view of force-displacement curves for forces up to 1 mN.

Supplementary Video:

1 In situ SEM nanoindentation – cyclic loading of the whole cleaned frustule

2 In situ SEM nanoindentation – cyclic loading of the upper cleaned valve

3 In situ SEM nanoindentation of a dried diatom cell 1

4 In situ SEM nanoindentation of a dried diatom cell 2

5 In situ SEM nanoindentation of a dried diatom cell 3

6 In situ SEM nanoindentation of a dried diatom cell 4

7 In situ SEM nanoindentation of a dried diatom cell 5

8 In situ SEM nanoindentation of a dried diatom cell 6

9 In situ SEM nanoindentation of a cleaned frustule 1

10 In situ SEM nanoindentation of a cleaned frustule 2

11 In situ SEM nanoindentation of a cleaned frustule 3

12 In situ SEM nanoindentation of a cleaned frustule 5

13 In situ SEM nanoindentation of a cleaned frustule 6
